# An Exploration of Adolescents’ Breakfast Skipping Focused on Gender Difference

**DOI:** 10.3390/nu18101487

**Published:** 2026-05-07

**Authors:** Jee Hye Lee

**Affiliations:** Department of Food Science and Nutrition, University of Ulsan, Ulsan 44610, Republic of Korea; leejh1@ulsan.ac.kr

**Keywords:** breakfast skipping, Korean adolescents, boys and girls, sociodemographic, dietary habit

## Abstract

**Background/Objectives**: Although breakfast skipping is on the rise worldwide, few have investigated the variables associated with breakfast skipping among adolescents with a focus on gender differences. This study aimed to examine the associations between health-related variables and breakfast skipping, and to investigate whether these associations differ by gender. **Methods**: Data from 54,653 adolescents who participated in Korean Youth Risk Behavior, a cross-sectional national survey conducted in 2024, were analyzed. Multivariable logistic regression was conducted to examine the variables associated with breakfast skipping. **Results**: In model 1, the main effects revealed that health-related variables were associated with skipping breakfast. In model 2, interaction effects were observed between gender and physical activity, as well as between gender and perceived health status. **Conclusions:** This study provides foundational evidence for developing nutrition education programs that consider gender differences.

## 1. Introduction

**Figure 1:**
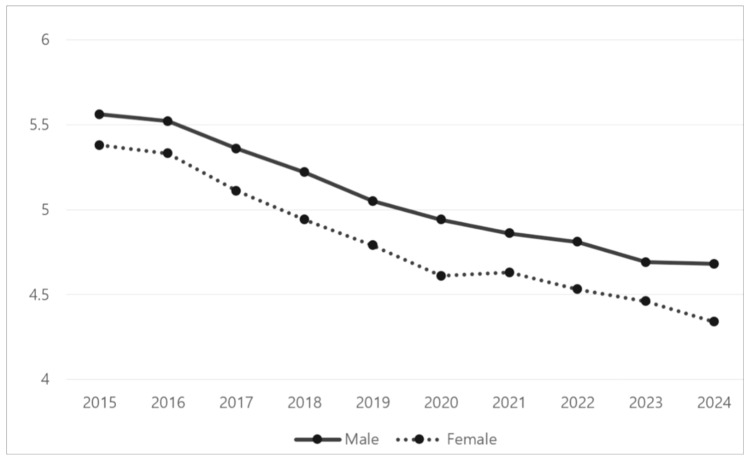
Trends in breakfast consumption among Korean adolescents (*X*-axis: year; *Y*-axis: breakfast frequency, times per week).

Breakfast is an important meal of the day [1], contributing daily nutrient intake of 20–35% of total daily energy [2] in the form of protein, iron, vitamin A, and vitamin C [3]. The health benefits of regular breakfast consumption have been linked to positive health behavior and stress control [4], healthy daytime cognitive functioning [5], appetite control and healthy body weight [6], and cardiometabolic profiles such as low LDL cholesterol [7]. On the other hand, breakfast skipping has been linked to risks of obesity [4], hypertension [8], and cardiovascular disease [7]. In adolescents specifically, breakfast skipping is associated with poor health-related behaviors [9], poor academic performance [10], and mental health problems [11]. Despite its correlations with poor health, the frequency of breakfast skipping among adolescents has increased. According to recent Korean School Health survey statistics, the rate of breakfast skipping among adolescents has increased by 4.78% every year, and everyday consumption has declined from 4.57 times in 2013 to 3.69 in 2022. The percentage of adolescents who skip breakfast more than five days a week has risen from 26.4% in 2013 to 42.45% in 2024 (Figure 1). Similar trends were reported among US high school students, whose regular breakfast consumption decreased from 38% in 2013 to 27% in 2023 [12]. In addition, 15 European countries have reported reductions in the rate of breakfast consumption [13].

Breakfast is an essential meal for adolescents, who need high nutrition levels to fuel the growth of their brains and bodies [14]. Adolescents undergo active physical and mental development on their way from childhood to adulthood [15], and they also experience the emergence of secondary sexual characteristics. The rapid physiological and psychological development that occurs in adolescence often brings changes to individuals’ pre-existing habits, so it is a good time to offer interventions that can establish healthy eating behaviors [16,17]. Furthermore, the dietary habits of adolescents are frequently carried into adult life [18,19]. Therefore, it is necessary to understand the factors underlying adolescents’ breakfast habits.

According to the literature, adolescents’ breakfast consumption cannot be explained by individual choice alone; it involves a combination of physical, mental, and dietary factors [20,21,22,23,24]. Physical health can be evaluated subjectively and objectively based on factors such as physical activity, sleep satisfaction, subjective body image, and perceived health status. For example, sleep timing, duration, and quality play an important part in the levels of counter-regulatory hormones (i.e., growth hormone or cortisol), leptin, and ghrelin, which are known to control appetite regulation [25]. Mental health is also a critical factor in adolescent eating behavior. During adolescence, psychological pressure from educational expectations and peer relationships tends to increase [26,27]. Factors such as stress, depression, and loneliness are known to be associated with hormone changes, irregular eating times, and fast-food consumption. Mental stress can lead to the consumption of low-quality food or irregular eating [24], including breakfast skipping, in adolescents. Furthermore, previous research revealed that unhealthy food patterns such as breakfast skipping are associated with other poor eating habits such as drinking sugar-sweetened beverages [28]. Therefore, it is important to study adolescents’ breakfast consumption from a complex structural perspective.

Little research has considered adolescents’ breakfast consumption with a focus on gender differences. Although several studies on gender differences in adolescents have been conducted [29], they have not considered breakfast consumption. Diet quality and eating behavior differences between men and women have been well documented in previous research and been shown to vary depending on biological characteristics such as genetics and psychosocial factors such as societal body ideals and parental eating styles [29,30]. For instance, health-protective foods (i.e., protein alternatives) are more widely consumed by men, whereas women tend to show more food restrictions (i.e., eating disorders) and poor emotional associations with food due to social pressures [31,32]. Additionally, gender differences in food acceptance and preferences were associated with body mass index (BMI) and overall dietary quality [32]. For instance, girls were more likely than boys to prefer fruits and vegetables, and boys were more likely than girls to choose meat, fish, poultry, and high-fat foods [33]. Consistent with the associations reported in previous studies, adolescent girls have been more likely than adolescent boys to skip breakfast during the past ten years, as shown in Figure 1. However, limited research has explored whether these associations differ by gender. Investigating gender differences in the associations between health-related factors and breakfast skipping is important for developing more targeted and effective health interventions.

This study uses data from the 2024 Korean Youth Risk Behavior web-based survey to examine the prevalence of breakfast consumption by Korean adolescents (controlling for sociodemographic characteristics) and find associations between health-related factors (physical health, mental health, and dietary habits) and breakfast skipping, and to determine whether these associations differ by gender.

## 2. Materials and Methods

### 2.1. Study Population

This study is based on data from the Korean Youth Risk Behavior Survey (KYRBWS), which was conducted in 2024 by the Korean Ministry of Education and Centers for Disease Control and Prevention. The KYRBWS is an anonymous, self-reported online survey to identify the health behavior of Korean middle and high school students. Specifically, the population was stratified by region (39 groups) and school type, resulting in 117 strata. A total of 400 middle schools and 400 high schools were allocated across 17 provinces, considering region, school type, and sex composition. Schools were randomly selected within each stratum, and one class per grade was randomly selected from each school; all students in selected classes were included, but long-term absence or inability to participate were excluded. The final analytic sample of the 20th (2024) KYRBWS comprised 54,653 students from 400 middle schools and 399 high schools in 17 cities (response rate = 94.9%); the sample was built using a stratified extraction method [34]. Sampling weights were applied to account for the complex survey design and to ensure national representativeness. All measurements in the Korea Youth Risk Behavior Survey are validated by expert panels [35]. This study was approved by the Institutional Review Board (2025R0017) of the University of Ulsan in Ulsan, Korea, and exempted from the requirement for informed consent because only public governmental data were analyzed.

### 2.2. Measures

As shown in Table 1, the measures analyzed in this study are sociodemographic variables (age, gender, school grade, BMI, economic status, academic performance, and residential status), physical health (physical activity, sleep satisfaction, subjective body image, and perceived health), mental health (sadness and hopeless and suicidal thoughts), and dietary behaviors (consumption of breakfast, sugar-sweetened beverages, and fast food).

#### 2.2.1. Sociodemographic Variables

The sociodemographic variables examined in this study were age, gender (girl or boy), school grade (middle school or high school), residential status (living with family or without family), academic performance, economic status (low, middle, or high), and BMI. BMI was computed as weight (kg) divided height squared (m^2^). Four groups were categorized based on criteria from the Korean Society for the Study of Obesity (2014): underweight (BMI < 18.5 kg/m^2^), normal weight (18.5–22.9 kg/m^2^), overweight (BMI ≥ 23 kg/m^2^), and obese (BMI ≥ 25 kg/m^2^).

#### 2.2.2. Physical Health

Physical activity was evaluated based on the question, “In the past 7 days, on how many days did you exercise hard or do cardio for more than 1 h?” and was answered with the number to days (0–7). Sleep satisfaction was assessed based on the question, “In the past week, did you sleep enough to recover from tiredness?” and was answered on a 5-point Likert scale from not enough at all (1) to very much enough (5). Subjective body image was evaluated based on the question, “What do you think about your body shape?” and was answered on a 5-point Likert scale (1 = very slim; 5 = very overweight). Perceived health status was assessed based on the question, “What do you think about your current health status?” and was answered on a 5-point Likert scale (1 = very healthy; 5 = very poor).

#### 2.2.3. Mental Health

Feelings of sadness and hopelessness were assessed based on the question, “Have you felt so sad or hopeless that you stopped your daily life for almost two weeks during past 12 months?” Suicidal thoughts were assessed based on the question, “During the past 12 months, have you ever had thoughts about suicide?” Both questions were answered either “yes” or “no.”

#### 2.2.4. Dietary Habits

The frequency of drinking sugar-sweetened beverages was assessed by asking, “In the past 7 days, how many days did you have sugar-sweetened beverages (carbonated beverages, energy drinks, sports drinks, fruit juice, coffee beverages, sweetened milk, etc.)?” The frequency of fast-food consumption was assessed using the question, “In the past 7 days, how many days did you have fast food (French fries, hamburger, etc.)?” Both those questions were answered with the following response options: (1) Not in the past 7 days, (2) once or twice, (3) 3–4 times, (5) 5–6 times, (6) every day, (7) twice a day, or (8) ≥3 times a day.

Breakfast consumption was assessed with the question, “In the past 7 days, how many days did you have breakfast (bread, porridge, cereal, etc.)?” and was answered using the following scale: (1) 0 days, (2) 1 day, (3) 2 days, (4) 3 days, (5) 4 days, (6) 5 days, (7) 6 days, or (8) 7 days. Breakfast consumption was recategorized into skipping and non-skipping based on previous studies [36,37]. Breakfast skipping has been defined in different ways in the literature review [38]. Among these, a criterion widely used in studies on adolescent breakfast consumption was adopted. Breakfast consumption less than five times per week (0–4 times) was classified as skipping, and eating breakfast five or more times per week was classified as non-skipping [37,39,40,41].

### 2.3. Statistical Analysis

The complex survey design was analyzed using IBM Statistical Package for Social Sciences version 29.0 (IBM Corp, Armonk, NY, USA). Statistical significance was assigned to *p*-values of less than 0.05. The sampling procedure followed three steps: population stratification, sample allocation, and sample extraction using a stratified cluster sampling method.

To examine the research objective, first, chi-square analyses were conducted to examine differences in breakfast skipping and non-skipping according to sociodemographic characteristics. Second, multivariable logistic regression analyses were conducted to identify the associations between (1) physical health variables (physical activity, sleep satisfaction, subjective body image, and perceived health status) and breakfast skipping, and to examine whether these associations differed by gender; (2) mental health variables (sadness and hopeless and suicidal thoughts) and breakfast skipping, and to examine whether these associations differed by gender; and (3) dietary habit variables (sugar-sweetened beverages consumption and fast-food consumption) and breakfast skipping, and to examine whether these associations differed by gender. Odds ratios (ORs) with 95% confidence intervals (CIs) are reported when adjusting for covariates. Gender was converted into a dummy variable (0 = male, 1 = female). Breakfast skipping was also dichotomized (0 = non-breakfast skipping, 1 = breakfast skipping). In all analysis, the lowest category was used as the reference group.

Model 1 included only the main effects of the independent variables, whereas model 2 included both the main effects and interaction terms between gender and health-related variables. All analyses were adjusted for sociodemographic characteristics of age, BMI, economic status, academic performance, and living apart from family. Those covariates were chosen based on previous research [42,43,44,45]. Prior to analysis, multicollinearity was assessed using the variance inflation factor (VIF), and no significant multicollinearity was detected (all VIFs < 5). Model fit was evaluated using the Hosmer–Lemeshow goodness-of-fit test (*p* > 0.05).

## 3. Results

### Frequency of Breakfast Skipping According to Sociodemographic Characteristics

The descriptive analysis presented in Table 2 shows significant differences in breakfast consumption according to participants’ characteristics. Breakfast consumption differed significantly by gender (χ^2^ = 155.418, *p* < 0.001). Boys were more likely to consume breakfast (27.0%) than to skip it (24.4%), whereas girls showed a higher proportion of breakfast skipping (25.7%) compared to non-skipping (22.9%). Age was also significantly associated with breakfast consumption (χ^2^ = 162.567, *p* < 0.001), with a general trend of increasing breakfast skipping as age increased. In particular, higher skipping proportions were observed among those aged 17 and 18. A significant difference was found by school grade (χ^2^ = 108.881, *p* < 0.001), with high school students more likely to skip breakfast than middle school students. Obesity status was significantly associated with breakfast consumption (χ^2^ = 107.559, *p* < 0.001), with a higher proportion of breakfast skipping observed among adolescents with obesity compared to those without. Economic status showed a significant association (χ^2^ = 128.542, *p* < 0.001). Adolescents with low economic status had a higher proportion of breakfast skipping, whereas those with high economic status were more likely to consume breakfast. Academic performance also differed significantly (χ^2^ = 775.777, *p* < 0.001), with students reporting lower academic performance showing a higher proportion of breakfast skipping compared to those with higher performance. Finally, residential status was significantly associated with breakfast consumption (χ^2^ = 78.940, *p* < 0.001).

**Figure 2:**
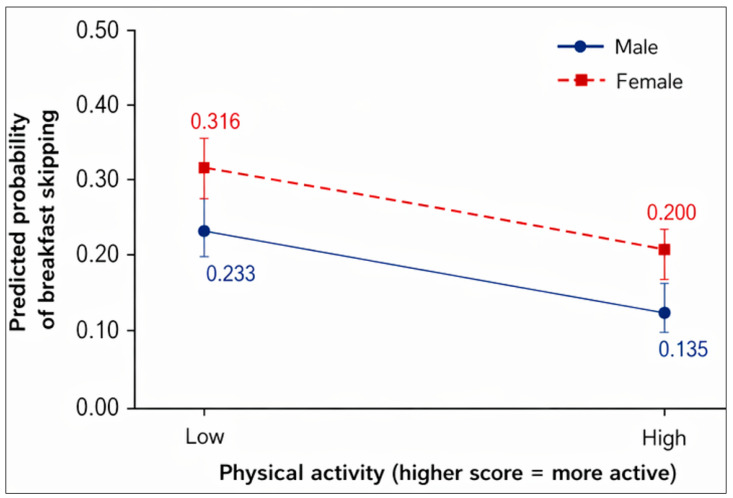
Interaction effect of gender on the association between physical activity and predicted probability of breakfast skipping.

**Figure 3:**
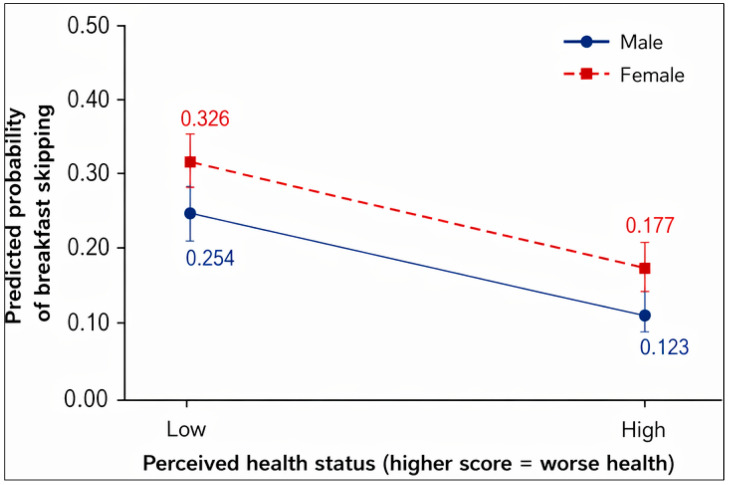
Interaction effect of gender on the association between perceived health status and probability of breakfast skipping.

Table 3 presents the results of the logistic regression analysis to examine the associations between breakfast skipping, gender, and variables reflecting physical health. In model 1, the ORs (95% CI) associated with breakfast skipping were 1.114 (1.064–1.166), *p* = 0.001 for gender; 1.021 (1.002–1.040), *p* = 0.001 for physical activity; 0.886 (0.870–0.901), *p* =0.001 for sleep satisfaction; 1.060 (1.031–1.090), *p* =0.001 for subjective body image; and 1.049 (1.028–1.071), *p* = 0.001 for perceived health status. In model 2, the ORs (95% CI) associated with breakfast skipping were 1.120 (1.070–1.172), *p* = 0.001 for gender; 0.946 (0.920–0.972), *p* = 0.001 for physical activity; 0.842 (0.796–0.891), *p* = 0.001 for sleep satisfaction; 1.031 (0.971–1.094), *p* = 0.001 for subjective body image; and 0. 317 (0.906–1.033), *p* = 0.324 for perceived health status. Interaction terms in model 2 showed 1.021 (1.002–1.040), *p* = 0.028 for gender × physical activity; 1.035 (0.998–1.073), *p* = 0.063 for gender × sleep satisfaction; 1.021 (0.986–1.057), *p* = 0.251 for gender × subjective body image; and 1.056 (1.012–1.102), *p* = 0.012 for gender × perceived health status. The interaction between gender and physical activity was statistically significant (Figure 2), indicating that the negative association between physical activity and breakfast skipping was observed in both boys and girls; however, the magnitude of this association attenuated in girls compared to boys. In addition, the association between perceived health and breakfast skipping differed by gender. Figure 3 illustrates that the increase in breakfast skipping with worse perceived health status was steeper in girls than in boys.

Table 4 presents the results of the logistic regression analysis to examine the associations between breakfast skipping, gender, and variables reflecting mental health. In model 1, the ORs (95% CI) associated with breakfast skipping were 1.217 (1.169–1.267), *p* = 0.001 for gender; 1.255 (1.202–1.309), *p* = 0.001 for sadness and hopelessness; and 1.009 (0.953–1.069), *p* = 0.749 for suicidal thoughts. In model 2, the ORs (95% CI) associated with breakfast skipping were 1.216 (1.168–1.266), *p* = 0.001 for gender; 1.219 (1.145–1.299), *p* = 0.001 for sadness and hopelessness; and 1.050 (0.963–1.144), *p* = 0.268 for suicidal thoughts. Interaction terms in model 2 showed 1.054 (0.968–1.149), *p* = 0.225 for gender × sadness and hopelessness and 0.935 (0.835–1.046), *p* = 0.240 for gender × suicidal thoughts. The interaction between gender and mental health variables was not statistically significant.

Table 5 presents the results of the logistic regression analysis to examine the associations between breakfast skipping, gender, and variables reflecting dietary habits. In model 1, the ORs (95% CI) associated with breakfast skipping were 1.268 (1.217–1.321), *p* = 0.001 for gender; 1.018 (1.013–1.023), *p* = 0.001 for sugar-sweetened beverages; and 1.026 (1.016–1.037), 0.001 for fast food. In model 2, the ORs (95% CI) associated with breakfast skipping were 1.275 (1.223–1.330), *p* = 0.001 for gender; 1.020 (1.013–1.026), *p* = 0.001 for sugar-sweetened beverages; and 1.021 (1.008–1.035), *p* = 0.002 for fast food. Interaction terms in model 2 showed 0.996 (0.986–1.006), *p* = 0.441 for gender × sugar-sweetened beverages and 1.012 (0.992–1.032), *p* = 0.235 for gender × fast food. The interaction between gender and dietary habit-related variables was not statistically significant.

## 4. Discussion

This study explores the association of health-related variables (physical health, mental health, and dietary habits) with breakfast skipping and determines whether these associations differ by gender. Model 1 evaluates the independent (main) effects of gender and health-related variables on breakfast skipping, while model 2 further assesses whether gender moderates these relationships by including interaction terms.

The findings for breakfast consumption frequency reveal similar patterns for boys and girls, particularly a tendency to decrease with age. Given Korea’s highly competitive university entrance environment, high school students are more likely than middle school students to get insufficient sleep [10], which can lead to later wake-up times. Higher breakfast consumption was observed among high academic performers of both genders than among middle and low academic performers. The relationship between better academic performance and breakfast skipping has been reported in several studies, and it has been explained that breakfast helps cognitive performance after night fasting [42,46]. In both genders, lower breakfast consumption was observed among those from low economic backgrounds than in those from middle- and high-status backgrounds, which is also consistent with prior research [47]. Breakfast skipping among low-income groups is not solely attributable to individual choice but originates from a complex structural problem involving economic disadvantages, time limitations, and inadequate access to food [48].

Consistent with previous research, lower breakfast consumption was found among overweight people than among normal-weight people. Overweight people might skip breakfast to limit their total energy, but skipping breakfast ultimately increases appetite and hunger, leading to overeating later in the day and diminished insulin sensitivity [43]. Thus, morning fasting leads to higher energy intake (i.e., free fatty acids) as compensation [49,50].

The findings of this study indicate that physical health, mental health, and dietary habits are associated with breakfast skipping in adolescents. Physical activity, sleep satisfaction, and subjective body image were associated with breakfast skipping in both boys and girls. The association between breakfast skipping and low physical exercise was explained in a previous study showing that high levels of physical activity contribute to strong eating regulation and eating behavior motivation [22]. The finding of an association between sleep satisfaction and breakfast skipping in both boys and girls is consistent with recent research showing that adolescents with later wake-up times or lower sleep quality were more likely than others to skip breakfast [51]. A reverse relationship has been suggested by circadian studies that showed that skipping breakfast, similar to other irregular meal timings, could affect peripheral circadian rhythms and sleep regulation [23]. The important role of breakfast regularity for adolescents has also been emphasized in a Japanese multi-dimensional sleep health study, which reported a relationship between favorable sleep timing and good subjective sleep health in adolescents [52].

Adolescents, both boys and girls, tended to skip breakfast more as they perceived their bodies to be more overweight. This finding is consistent with prior research that showed a relationship between body image and eating patterns among adolescents [21]. That research found that overweight boys and girls who were dissatisfied with their perceived body image and preferred a thinner figure were likely to eat fewer than three meals per day, to skip breakfast, and to eat a lower-than-average diversity of food. In this study, the difference in perceived health status by gender is meaningful: perceived health status showed significant associations with breakfast skipping only among adolescent girls, with perceived good health associated with a lower likelihood of skipping breakfast. Although prior studies reported that self-rated health rarely correlates with breakfast skipping, one previous study suggested that girls and women have healthier breakfast dietary habits and this may be because they are more careful about their body image and dieting than boys and men [10]. The multivariable-adjusted model 1 in this study shows that adolescent boys and girls who felt sad and hopeless were associated with a higher likelihood of breakfast skipping than those who did not, but those who had not thought about suicide were associated with a lower likelihood of breakfast skipping than those who had. The negative correlation between breakfast skipping and mental health can be attributed to glucose intake [53] and hormone levels in adolescents [54]. Nighttime fasting lowers glucose levels in the blood, which results in the release of cortisol [55], and this is related to irritable feelings and anxiety. When breakfast is eaten, glucose converted from the meal reduces high cortisol levels, which is linked to positive mood and lower stress and depressive feelings [56,57]. That explanation is consistent with the findings that adolescents who eat breakfast regularly reported lower levels of feelings of sadness and hopelessness than those who regularly skip breakfast.

Finally, adolescents of both genders who frequently consumed sugar-sweetened beverages (SSBs) and fast food were more likely than those who did not skip breakfast. Despite limited research into the association between breakfast skipping and unhealthy eating behaviors among adolescents, our findings are consistent with previous studies demonstrating associations between unhealthy eating behaviors and the consumption of SSBs [20] and fast food [58]. For instance, in a study of women who were university students in Saudi Arabia, a low frequency of junk food and soft drink consumption was the strongest predictor of eating breakfast at home [52]. Although many studies have reported that consuming junk food and SSBs has a direct influence on breakfast skipping, little research has provided a clear explanation for that association. One proposed reason lies in the correlation between home breakfast skipping and insufficient sleep due to high consumption of caffeine and high screen time among Saudi Arabian university students [20].

The results of the moderating effects in Model 2 highlight the importance of under-standing gender differences in eating behaviors. Specifically, the first finding showed that higher levels of physical activity were associated with a lower probability of breakfast skipping for both males and females; however, this effect was stronger in boys than in girls. Previous studies have shown that males often exhibit higher levels of physical ac-tivity than females [59]. In addition, males tend to associate healthy eating behaviors with physical activity, whereas females are more likely to engage in physical activity for psychological reasons such as weight control [30,32,60]. The second finding indicated that poorer perceived health status was associated with higher probability of breakfast skip-ping among both males and females, but this association was stronger in females. Pre-vious studies have found that females usually show lower levels of perceived health status [61]. This finding implies that negative perceptions of health status among girls are more likely to contribute to behavioral changes toward healthier eating behaviors. In contrast, boys appear to have a more limited tendency to associate perceived health status with eating behaviors. Therefore, these findings suggest the moderating role of gender should be considered in studies exploring the link between breakfast skipping and physical health-related variables.

This study makes several academic and practical contributions. The use of nationally surveyed KYRBWS data ensures the representativeness of the findings. Although gender differences in eating behavior have been studied [62,63], comparative research of adolescent breakfast skipping by gender has been lacking. This study broadens the existing literature on adolescents’ dietary habits by providing evidence of an association between breakfast consumption and various physical and mental health factors. Accounting for potential confounding sociodemographic factors enhances the validity of our findings. In line with previous studies, the results presented here suggest that an integrated approach incorporating physical health, mental health, and dietary habits can enhance the effectiveness of school nutrition intervention programs.

## 5. Limitations

Although the KYRBWS data were collected through an anonymous online survey, potential biases, such as recall or social desirability [64], due to a lack of comprehension of behavior or normative pressure, are acknowledged. Specifically, the underestimation of BMI calculated from self-reported height and weight should be considered [65]. Future research using directly measured data analysis is recommended. Also, as this study is based on a secondary analysis of cross-sectional data, causal relationships cannot be established, and the observed associations should be interpreted with caution. Even though this study reflects a national sample of adolescents, it is limited to Korean adolescents. Future empirical studies need to use follow-up cohort studies with broader age samples and muti-country comparisons. In this study, breakfast consumption was assessed using a frequency-based question. The use of 24 h dietary recall techniques in future studies could enable an analysis of energy and micronutrient intake.

## 6. Conclusions

Our findings provide evidence that the frequency of Korean adolescents’ breakfast consumption differs according to their sociodemographic characteristics. Also, they suggest that adolescents’ breakfast skipping should be analyzed using a broad approach that considers physical health, mental health, and dietary habits. It is especially important to use different approaches for adolescent boys and girls. This study confirms previously reported associations between breakfast skipping and physical health, mental health, and dietary habits among adolescent boys and girls. A unique finding of this study is that this study found significant interaction effects between gender and health-related factors. It indicates that the relationship between breakfast skipping and health-related factors differs between adolescent boys and girls after accounting for interaction effects. The critical role of regular breakfast consumption in adolescent health is already widely known, but gender differences have received little previous attention. The environments surrounding adolescents are changing rapidly, so a multifaceted approach is required to accurately analyze food-related behavior. Thus, considering physical health, mental health, and dietary factors can contribute to the development of policies that improve adolescents’ health behavior and to effective nutrition interventions that can be applied differently based on gender.

## Figures and Tables

**Table 1:** Questions and measurement scale.

Variable	Question	Measurement Scale
***Sociodemographic***		
Gender ^2^	What is your gender?	(0) Boys (1) Girls
Age ^2^	What is your age?	____ years old
School grade ^2^	What is your current school level?	(1) Middle school (2) High school
BMI ^2^	kg/m^2^	(1) Underweight (2) Normal (3) Overweight (4) Obesity
Economic status ^2^	What is your family’s economic status	(1) Low (2) Middle (3) High
Academic performance ^2^	How would you rate your school grades over the past year?	(1) Low (2) Middle (3) High
Residential status ^2^	What is your current type of residence?	(0) Living with family (1) Living apart from family
***Physical health***		
Physical activity ^1^	In the last 7 days, on how many days did you exercise hard or do cardio for more than 1 h?	(1) 0 days (2) 1 day (3) 2 days (4) 3 days (5) 4 days (6) 5 days (7) 6 days (8) 7 days
Sleep satisfaction ^1^	In the last week, did you sleep enough to recover from tiredness?	(1) Not enough at all (2) Not enough (3) Fair (4) Enough (5) Very enough
Subjective body image ^1^	What do you think about your body shape?	(1) Very slim (2) Slim (3) Average (4) Overweight (5) Very overweight
Perceived health status ^1^	What do you think about your current health status?	(1) Very healthy (2) Good (3) Fair (4) Poor (5) Very poor
***Mental health***		
Sadness and hopeless ^1^	Have you felt so sad and hopeless that you stopped your daily life for almost two weeks during past 12 months?	(1) No (2) Yes
Suicidal thoughts ^1^	During the past 12 months, have you ever had thoughts about suicide?	(1) No (2) Yes
***Dietary habits***		
Breakfast consumption ^1^	In the past 7 days, how many days did you have breakfast (including bread, porridge, cereal, etc.)?	(1) 0 days (2) 1 day (3) 2 days (4) 3 days (5) 4 days (6) 5 days (7) 6 days (8) 7 days
Sugar-sweetened beverages consumption ^1^	In the past 7 days, how many days did you have sugar-sweetened beverages (including carbonated beverages, energy drinks, sports drinks, fruit juice, coffee beverage, sweetened milk, etc.)?	(1) Not in the last 7 days (2) once or twice a week (3) 3–4 times a week (5) 5–6 times a week (6) Every day (7) Twice a day (8) ≥3 times a week
Fast-food consumption ^1^	In the past 7 days, how many days did you have fast food (French fries, hamburger, etc.)?	(1) Not in the last 7 days (2) Once or twice a week (3) 3–4 times a week (5) 5–6 times a week (6) Every day (7) Twice a day (8) ≥3 times a week

Note: Superscript ^1^ indicates continuous variables, and superscript ^2^ indicates categorical variables.

**Table 2:** Descriptive analysis of breakfast eaters and skippers.

		Breakfast Consumption, n (%)			χ^2^ (*p*)
Variables		Non-Skipping	Skipping	Total	
Gender	Boys	14,752 (27.0)	13,338 (24.4)	28,090 (51.4)	155.418 ***
	Girls	12,533 (22.9)	14,030 (25.7)	26,563 (48.6)	
Age	14	5330 (9.8)	4567 (8.4)	9897 (18.1)	162.567 ***
	15	4770 (8.7)	4667 (8.5)	9437 (17.3)	
	16	4497 (8.2)	4773 (8.7)	9270 (17.0)	
	17	4279 (7.8)	4748 (8.7)	9027 (16.5)	
	18	3864 (7.1)	4317 (7.9)	8181 (15.0)	
	19	1927 (3.5)	2099 (3.8)	4026 (7.4)	
School grade	Middle school	15,130 (27.7)	13,957 (25.5)	29,087 (53.2)	108.881 ***
	High school	12,155 (22.2)	13,411 (24.5)	25,566 (46.8)	
Obesity	No	21,810 (42)	21,103 (40.6)	42,913 (82.6)	107.559 ***
	Yes	4229 (8.1)	4787 (9.3)	9016 (17.4)	
Economic status	Low	440 (0.8)	655 (1.2)	1095 (2.0)	128.542 ***
	Middle	23,324 (42.7)	23,900 (43.7)	47,224 (86.4)	
	High	3519 (6.4)	2810 (5.1)	6329 (11.6)	
Academic performance	Low	2002 (3.7)	3290 (6.0)	5292 (9.6)	775.777 ***
	Middle	20,875 (38.2)	21,456 (39.3)	42,331 (77.5)	
	High	4407 (8.1)	2620 (4.8)	7027 (12.9)	
Residential status	Living with family	25,718 (47.1)	26,243 (48.0)	51,961 (95.1)	78.940 ***
	Living apart from family	1565 (2.9)	1120 (2.0)	2685 (4.9)	

*** *p* < 0.001.

**Table 3:** Logistic regression model examining interaction effects of gender and physical health on breakfast skipping.

Variables	Model 1 †		Model 2 ‡	
	OR ^a^ (95% CI)	*p*-Value	OR ^a^ (95% CI)	*p*-Value
***Main effects***				
Gender	1.114 (1.064–1.166)	0.001	1.120 (1.070–1.172)	0.001
Physical activity	0.974 (0.965–0.983)	0.001	0.946 (0.920–0.972)	0.001
Sleep satisfaction	0.886 (0.870–0.901)	0.001	0.842 (0.796–0.891)	0.001
Subjective body image	1.060 (1.031–1.090)	0.001	1.031 (0.971–1.094)	0.317
Perceived health status	1.049 (1.028–1.071)	0.001	0.967 (0.906–1.033)	0.324
***Interaction effects***				
Gender × physical activity			1.021 (1.002–1.040)	0.028
Gender × sleep satisfaction			1.035 (0.998–1.073)	0.063
Gender × subjective body image			1.021 (0.986–1.057)	0.251
Gender × perceived health status			1.056 (1.012–1.102)	0.012

All estimates were obtained by accounting for the stratified cluster sampling design. All models were adjusted for sociodemographic variables, including age, BMI, grade, economic status, academic performance, and living status. ^a^ Odds ratio (95% confidence interval). † Model 1 included only the main effects of all variables; ‡ Model 2 included both main effects and all two-way interaction effects.

**Table 4:** Logistic regression model examining interaction effects of gender and mental health on breakfast skipping.

Variables	Model 1 †	Model 2 ‡		
	OR ^a^ (95% CI)	*p*-Value	OR ^a^ (95% CI)	*p*-Value
***Main effects***				
Gender	1.217 (1.169–1.267)	0.001	1.216 (1.168–1.266)	0.001
Sadness and hopelessness	1.255 (1.202–1.309)	0.001	1.219 (1.145–1.299)	0.001
Suicidal thoughts	1.009 (0.953–1.069)	0.749	1.050 (0.963–1.144)	0.268
***Interaction effects***				
Gender × sadness & hopelessness			1.054 (0.968–1.149)	0.225
Gender × suicidal thoughts			0.935 (0.835–1.046)	0.240

All estimates were obtained by accounting for the stratified cluster sampling design. All models were adjusted for sociodemographic variables, including age, BMI, grade, economic status, academic performance, and living status. ^a^ Odds ratio (95% confidence interval); † Model 1 included only the main effects of all variables. ‡ Model 2 included both main effects and all two-way interaction effects.

**Table 5:** Logistic regression model examining interaction effects of gender and dietary habits on breakfast skipping.

Variables	Model 1 †		Model 2 ‡	
	OR ^a^ (95% CI)	*p*-Value	OR ^a^ (95% CI)	*p*-Value
***Main effects***				
Gender	1.268 (1.217–1.321)	0.001	1.275 (1.223–1.330)	0.001
Sugar-sweetened beverages	1.018 (1.013–1.023)	0.001	1.020 (1.013–1.026)	0.001
Fast food	1.026 (1.016–1.037)	0.001	1.021 (1.008–1.035)	0.002
***Interaction effects***				
Gender × sugar-sweetened beverages			0.996 (0.986–1.006)	0.441
Gender × fast food			1.012 (0.992–1.032)	0.235

All estimates were obtained by accounting for the stratified cluster sampling design. All models were adjusted for sociodemographic variables, including age, BMI, grade, economic status, academic performance, and living status. ^a^ Odds ratio (95% confidence interval); † Model 1 included only the main effects of all variables. ‡ Model 2 included both main effects and all two-way interaction effects.

## Data Availability

The data that support the findings of this study are available upon reasonable request.
